# Association of Prepubertal and Adolescent Androgen Concentrations With Timing of Breast Development and Family History of Breast Cancer

**DOI:** 10.1001/jamanetworkopen.2019.0083

**Published:** 2019-02-22

**Authors:** Lauren C. Houghton, Julia A. Knight, Ying Wei, Russell D. Romeo, Mandy Goldberg, Irene L. Andrulis, Angela R. Bradbury, Saundra S. Buys, Mary B. Daly, Esther M. John, Wendy K. Chung, Regina M. Santella, Frank Z. Stanczyk, Mary Beth Terry

**Affiliations:** 1 Department of Epidemiology, Columbia University Mailman School of Public Health, New York, New York; 2 Division of Epidemiology, Dalla Lana School of Public Health, University of Toronto, Toronto, Ontario, Canada; 3 Lunenfeld–Tanenbaum Research Institute, Sinai Health System, Toronto, Ontario, Canada; 4 Department of Biostatistics, Columbia University Mailman School of Public Health, New York, New York; 5 Psychology and the Neuroscience and Behavior Program, Barnard College of Columbia University, New York, New York; 6 Department of Molecular Genetics, University of Toronto, Toronto, Ontario, Canada; 7 Department of Medical Ethics and Health Policy, Perelman School of Medicine, University of Pennsylvania, Philadelphia; 8 Division of Hematology/Oncology, Department of Medicine, The Perelman School of Medicine of the University of Pennsylvania, Philadelphia; 9 Department of Medicine, University of Utah Health Sciences Center, Huntsman Cancer Institute, Salt Lake City; 10 Department of Clinical Genetics, Fox Chase Cancer Center, Philadelphia, Pennsylvania; 11 Division of Oncology, Department of Medicine, Stanford University School of Medicine, Stanford, California; 12 Stanford Cancer Institute, Stanford University School of Medicine, Stanford, California; 13 Herbert Irving Comprehensive Cancer Center, Columbia University Medical Center, New York, New York; 14 Department of Pediatrics, Columbia University Medical Center, New York, New York; 15 Department of Medicine, Columbia University Medical Center, New York, New York; 16 Environmental Health Sciences, Columbia University Mailman School of Public Health, New York, New York; 17 Department of Obstetrics and Gynecology, Keck School of Medicine, University of Southern California, Los Angeles; 18 Department of Preventive Medicine, Keck School of Medicine, University of Southern California, Los Angeles

## Abstract

**Importance:**

Early breast development is a risk factor for breast cancer, and girls with a breast cancer family history (BCFH) experience breast development earlier than girls without a BCFH.

**Objectives:**

To assess whether prepubertal androgen concentrations are associated with timing of breast development (analysis 1) and to compare serum androgen concentrations in girls with and without a BCFH (analysis 2).

**Design, Setting, and Participants:**

Prospective cohort study of 104 girls aged 6 to 13 years at baseline using data collected between August 16, 2011, and March 24, 2016, from the Lessons in Epidemiology and Genetics of Adult Cancer From Youth (LEGACY) Girls Study, New York site.

**Exposures:**

Analysis 1 included serum concentrations of dehydroepiandrosterone sulfate, androstenedione, and testosterone (free and total) measured before breast development and divided at the median into high and low categories. Analysis 2 included the degree of BCFH: first-degree was defined as having a mother with breast cancer and second-degree was defined as having a grandmother or aunt with breast cancer.

**Main Outcomes and Measures:**

Analysis 1 included age at onset of breast development measured using the Pubertal Development Scale (scores range from 1-4; scores ≥2 indicate breast development), and analysis 2 included serum androgen concentrations. We also assessed breast cancer–specific distress using the 8-item Child Impact of Events Scale.

**Results:**

Our analyses included 36 girls for the prospective model, 92 girls for the cross-sectional model, and 104 girls for the longitudinal model. Of the 104 girls, the mean (SD) age at baseline was 10.3 (2.5) years, and 41 (39.4%) were non-Hispanic white, 41 (39.4%) were Hispanic, 13 (12.5%) were non-Hispanic black, and 9 (8.7%) were other race/ethnicity. Forty-two girls (40.4%) had a positive BCFH. Girls with prepubertal androstenedione concentrations above the median began breast development 1.5 years earlier than girls with concentrations below the median (Weibull survival model–estimated median age, 9.4 [95% CI, 9.0-9.8] years vs 10.9 [95% CI, 10.4-11.5] years; *P* = .001). Similar patterns were observed for dehydroepiandrosterone sulfate (1.1 years earlier: age, 9.6 [95% CI, 9.1-10.1] years vs 10.7 [95% CI, 10.2-11.3] years; *P* = .009), total testosterone (1.4 years earlier: age, 9.5 [95% CI, 9.1-9.9] years vs 10.9 [95% CI, 10.4-11.5] years; *P* = .001), and free testosterone (1.1 years earlier: age, 9.7 [95% CI, 9.2-10.1] years vs 10.8 [95% CI, 10.2-11.4] years; *P* = .01). Compared with girls without BCFH, girls with a first-degree BCFH, but not a second-degree BCFH, had 240% higher androstenedione concentrations (geometric means: no BCFH, 0.49 ng/mL vs first-degree BCFH, 1.8 ng/mL vs second-degree, 1.6 ng/mL; *P* = .01), 10% higher total testosterone concentrations (12.7 ng/dL vs 14.0 ng/dL vs 13.7 ng/dL; *P* = .01), and 92% higher free testosterone concentrations (1.3 pg/mL vs 2.5 pg/mL vs 0.3 pg/mL; *P* = .14). The dehydroepiandrosterone sulfate concentration did not differ between BCFH-positive and BCFH-negative girls but was elevated in girls with breast cancer–specific distress.

**Conclusions and Relevance:**

Our findings suggest that androgen concentrations may differ between girls with and without a BCFH and that elevated hormone concentrations during adolescence may be another factor to help explain the familial clustering of breast cancer.

## Key Points

**Question:**

Are serum concentrations of androgens in prepubertal girls associated with a family history of breast cancer?

**Findings:**

In this cohort study of 104 prepubertal girls, total testosterone concentrations were 10% higher, free testosterone concentrations were 92% higher, and androstenedione concentrations were 240% higher in girls with first-degree, but not second-degree, relatives with breast cancer compared with girls without a family history of breast cancer. Findings were statistically significant for androstenedione and total testosterone concentrations, but findings for free testosterone concentrations were not statistically significant.

**Meaning:**

If replicated in larger studies, these results may support the finding that elevated androgen concentrations in adolescent girls whose mother has breast cancer may be another factor contributing to the familial clustering of breast cancer.

## Teaser

This cohort study evaluates the association of prepubertal androgen concentrations with timing of breast development in girls with or without a family history of breast cancer.

## Introduction

A large body of epidemiologic research supports the role of estrogen in postmenopausal breast cancer^1^; however, the role of androgens (metabolic precursors to estrogens) in breast cancer risk has received less attention. Higher concentrations of prediagnostic androgens are associated with both premenopausal and postmenopausal breast cancer.^2,3^ In a nested case-control study of 1375 postmenopausal women, testosterone concentrations were associated with a 55% increased risk of postmenopausal breast cancer among women in the top quartile vs bottom quartile of concentrations.^2^ In the large European Prospective Investigation Into Cancer and Nutrition study, androgens have been associated with a 30% to 55% increase in premenopausal breast cancer risk.^3^ During prepubescence, androgen concentrations begin to rise between ages 6 to 8 years, and a study^4^ reported that girls with elevated androgen concentrations had earlier onset of breast development. We recently reported a 50% higher rate of earlier breast development in girls with a breast cancer family history (BCFH) compared with girls without a family history (no BCFH).^5^ Early breast development, independent of age at menarche, increases the risk of breast cancer by 20%.^6^

If and when androgen trajectories can be modified is unknown. In one study,^7^ androgen concentrations in adolescent girls with late menarche were either above or below the range of androgen concentrations in girls with early and typical age at menarche. This finding suggests that varying factors in girls with late menarche influence adolescent androgen production. First, these girls may have had early thelarche. Early thelarche is associated with obesity^8^; thus, childhood body mass index trajectories may be implicated. Stress reactivity may also play a role.^9,10,11^ Stress reactivity is sensitive to modulation during puberty^12,13^; therefore, differences between hyperreactivity and hyporeactivity may explain the wide range in androgen hormone concentrations in girls. Given that stressful conditions have been shown to alter pubertal timing^10^—and specifically at the family level, that a father’s absence accelerates pubertal timing^14^—breast cancer in mothers may also pose a similar stressful childhood environment.

We hypothesized that the association between elevated adult androgen concentrations and increased breast cancer risk is programmed during puberty and modified by a BCFH, which represents both shared environment and shared underlying genetic susceptibility. Thus, we examined whether prepubertal concentrations of androgens were associated with breast development in girls with and without a BCFH and whether BCFH was associated with androgen concentrations. If androgen concentrations are different in girls with and without a BCFH, this finding may support the hypothesis that additional factors contribute to familial clustering of breast cancer.

## Methods

Our reporting adheres to the Strengthening the Reporting of Observational Studies in Epidemiology (STROBE) reporting guideline for cohort studies. Using the New York site of the Lessons in Epidemiology and Genetics of Adult Cancer From Youth (LEGACY) Girls Study, a prospective cohort, we examined girls aged 6 to 13 years at baseline and monitored them longitudinally for pubertal development.^15^ We established the cohort in 2011 with continued follow-up. We conducted the present analysis in 2017 using data collected between August 16, 2011, and March 24, 2016. Every 6 months, girls and their mothers had follow-up visits involving anthropometric measurements (height and weight to calculate body mass index) and pubertal development assessment. Mothers reported the race/ethnicity of their daughters. We collected blood samples annually. We assessed breast development using the Pubertal Development Scale; scores range from 1 to 4, and scores of 2 or higher indicate breast development. We obtained institutional review board approval from Columbia University, New York, New York, to conduct the study. Mothers or guardians provided written informed consent, and girls provided assent based on institutional standards.

We measured 3 androgen concentrations in serum at the Reproductive Endocrine Research Laboratory at the Keck School of Medicine, Los Angeles, California, by using radioimmunoassays with preceding organic solvent extraction and celite column partition chromatography^16^ to measure testosterone and androstenedione concentrations. Dehydroepiandrosterone sulfate (DHEA-S) and sex hormone-binding globulin were measured by direct chemiluminescent immunoassay. We calculated the free testosterone concentration using a validated algorithm^17^ that requires total testosterone and sex hormone-binding globulin concentrations. The within-batch coefficients of variation ranged from 6% to 13%, and the between-batch coefficients of variation ranged from 5% to 7%. All androgen concentrations were log-transformed to improve normality. The precursor to the other androgens, DHEA-S, was correlated with androstenedione (*r* = 0.65), total testosterone (*r* = 0.62), and free testosterone (*r* = 0.71).

We classified girls as BCFH-positive if the participating mother or guardian reported a BCFH in the daughter’s first- or second-degree relatives. First-degree BCFH is having a mother with breast cancer, and second-degree BCFH is having a grandmother or aunt with breast cancer. In addition to the categorical BCFH variable, we used the Breast and Ovarian Analysis of Disease Incidence and Carrier Estimation Algorithm (BOADICEA) risk model to estimate lifetime breast cancer risk for each girl^18^ by estimating the absolute risk based on family pedigree information. The model produces a continuous score ranging from 0 to 100 for lifetime and fixed intervals of risk. We used the scores to rank the girls according to their risk of breast cancer and to provide greater precision. The scores cannot be interpreted as absolute risks because BOADICEA has been validated only for use in adult women.^19^

We evaluated breast cancer–specific distress at the baseline visits and at follow-up visits 1, 3, and 5 using the 8-item Child Impact of Events Scale, a developmentally appropriate version of the Revised Impact of Event Scale.^20,21,22^ Both scales evaluate intrusion and avoidance as indices of breast cancer–specific distress.^23,24^ We summed the scores from the intrusion and avoidance indices to create a total distress score. Potential scores for the total Child Impact of Events Scale can range from 0 to 40, and a higher score indicates higher breast cancer–specific distress. When we examined the association of breast cancer-specific distress and the age of breast development, we collapsed the individual visit scores into 1 binary variable (yes vs no). When we examined the association between androgen concentrations by BCFH, we used the score at the same study visit as when we collected the serum sample; if that score was missing, we used the score from the preceding visit.

### Statistical Analysis

We conducted 2 primary analyses, which included an examination of whether prepubertal androgens were associated with earlier breast development (analysis 1) and a comparison of serum androgen concentrations in girls with and without a BCFH (analysis 2).

For analysis 1, we used parametric Weibull survival models to estimate the median (95% CI) age at onset of breast development in girls with higher (above the median) vs lower (below the median) androgen concentrations measured at the same study visit as pubertal assessment for the cross-sectional analysis (n = 92) and before any breast development for the prospective analysis (n = 36). We adjusted our main model (model 1) for an interaction between androgen concentration and age (at sample collection), race/ethnicity, and body mass index. Subsequent models included model 1 variables with the BOADICEA risk score (model 2) and model 1 variables with breast cancer–specific distress (model 3).

For analysis 2, a longitudinal analysis using mixed models clustered on the individual, we compared the mean androgen concentrations by degree of BCFH (first-degree relatives vs BCFH-negative and second-degree relatives vs BCFH-negative) after controlling for age and body mass index. We then adjusted the model for breast cancer–specific distress and also ran a model with breast cancer–specific distress without BCFH. Analyses included 104 girls with repeated blood collection samples (mean [range], 2.4 [1-5] samples).

## Results

Our analyses included 36 girls for the prospective model, 92 girls for the cross-sectional model, and 104 girls for the longitudinal model. Of the 104 girls, the mean (SD) age was 10.3 (2.5) years, and the distribution of race/ethnicity was as follows: 41 (39.4%) non-Hispanic white, 41 (39.4%) Hispanic, 13 (12.5%) non-Hispanic black, and 9 (8.7%) other race/ethnicity (Table 1). Most of the mothers (67 [64.4%]) held a bachelor’s or graduate degree, and 42 girls (40.4%) had a positive BCFH (Table 1).

**Table 1.** Hormone Concentrations by Baseline Characteristics for 104 Participants of the LEGACY Girls Study, New York Site

Characteristic	Participants, No. (%)	Hormone Concentrations, Median (Interquartile Range)			
		DHEA-S, μg/dL	Androstenedione, ng/mL	Total Testosterone, ng/dL	Free Testosterone, pg/mL
Age, y					
6-8	35 (33.7)	24.8 (20.5-44.1)	0.1 (0.9-0.2)	4.2 (2.6-5.9)	0.5 (0.3-1.0)
9-11	39 (37.5)	67.2 (34.9-105.0)	0.4 (0.2-0.5)	9.2 (7.2-14.9)	1.7 (1.1-2.6)
12-16	30 (28.9)	78.2 (62.8-157.0)	0.8 (0.6-1.0)	22.0 (16.7-31.1)	4.0 (2.9-6.4)
Race/ethnicity					
Hispanic	41 (39.4)	61.8 (26.9-92.6)	0.4 (0.2-0.8)	11.3 (5.8-26.7)	1.8 (0.8-4.6)
Non-Hispanic black	13 (12.5)	68.9 (27.2-94.8)	0.2 (0.9-0.5)	6.2 (2.8-11.9)	1.0 (0.4-2.6)
Non-Hispanic white	41 (39.4)	44.3 (28.5-81.1)	0.4 (0.2-0.6)	8.8 (4.9-18.0)	1.3 (0.8-2.6)
Other^a^	9 (8.7)	78.6 (62.1-108.0)	0.4 (0.4-0.6)	13.0 (8.6-18.3)	2.6 (1.3-3.8)
Maternal educational level					
Some college or less	37 (35.6)	68.2 (22.4-102.0)	0.4 (0.1-0.6)	9.5 (2.8-18.8)	1.7 (0.5-3.8)
Bachelor’s degree	29 (27.9)	69.1 (38.3-105.0)	0.4 (0.2-0.7)	11.3 (5.9-25.6)	2.2 (0.8-4.1)
Graduate degree	38 (36.5)	50.6 (29.9-72.2)	0.4 (0.2-0.6)	7.9 (5.5-15.9)	1.4 (0.6-2.6)
Body mass index^b^					
<85th Percentile	75 (72.8)	51.6 (22.4-80.6)	0.4 (0.2-0.7)	10.0 (4.6-18.8)	1.7 (0.7-3.0)
≥85th Percentile	28 (27.2)	76.7 (48.1-131.0)	0.3 (0.2-0.6)	7.9 (6.0-20.2)	1.7 (1.2-4.6)
Breast cancer family history					
Negative	62 (59.6)	52 (22.4-75.3)	0.3 (0.1-0.5)	7.3 (4.1-13.3)	1.3 (0.5-2.5)
Positive	42 (40.4)	72.4 (36.4-110.0)	0.5 (0.2-0.9)	15.4 (7.5-26.7)	2.6 (1.2-6.2)

Abbreviations: DHEA-S, dehydroepiandrosterone sulfate; LEGACY, Lessons in Epidemiology and Genetics of Adult Cancer From Youth.

SI conversion factors: To convert DHEA-S to micromoles per liter, multiply by 0.027; androstenedione to nanomoles per liter, multiply by 0.0349; total testosterone to nanomoles per liter, multiply by 0.0347; and free testosterone to picomoles per liter, multiply by 3.47.

^a^ Other race/ethnicity includes Asian and other.

^b^ Data on body mass index were missing for 1 patient.

**Figure 1.**
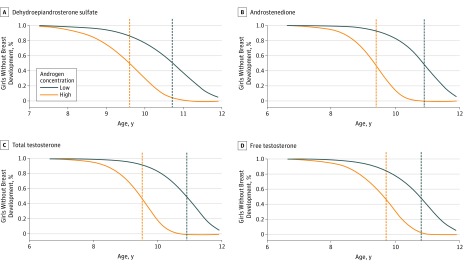
Estimated Density Curves for Age at Onset of Breast Development by Androgen Concentrations in the Lessons in Epidemiology and Genetics of Adult Cancer From Youth (LEGACY) Girls Study, New York Site Median (95% CI) ages were generated from an interval-censored Weibull regression model using a dichotomous variable based on the median concentration for each androgen and adjusted for race/ethnicity, body mass index, and an interaction term between androgen concentration and age at sample collection. Curves and medians pictured result from the prospective analysis on the subcohort of 36 girls in whom the androgen concentration was measured before any breast development.

Breast development occurred earlier in girls with elevated concentrations of prepubertal androgens. Specifically, girls with higher androstenedione concentrations began breast development 1.5 years earlier (median [95% CI] age, 9.4 [9.0-9.8] years) than girls with lower androstenedione concentrations (10.9 [10.4-11.5] years) (*P* = .001). Similar patterns were observed, with breast development occurring 1.1 years earlier (DHEA-S: median [95% CI] age, 9.6 [9.1-10.1] vs 10.7 [10.2-11.3] years; *P* = .009), 1.4 years earlier (total testosterone: 10.9 [10.4-11.5 ] vs 9.5 [9.1-9.9] years; *P* = .001), and 1.1 years earlier (free testosterone: 10.8 [10.2-11.4 ] vs 9.7 [9.2-10.1] years; *P* = .01) for higher compared with lower androgen concentrations (Figure 1). Positive associations between androstenedione, total testosterone, and free testosterone concentrations and early age at thelarche were evident after adjustment for family breast cancer risk as measured by the BOADICEA (Table 2). Family history score by this algorithm was also positively associated with earlier age at thelarche (model 2 in Table 2). Breast cancer–specific distress did not attenuate the association between androgen concentrations and the timing of breast development (model 3 in Table 2). We also calculated the medians (95% CIs) from the larger cross-sectional cohort (n = 92), in whom we assessed androgen concentrations and pubertal development stage concurrently. Breast development occurred 0.7 years earlier in girls with higher androstenedione concentrations (*P* = .30), 0.5 years earlier with higher total testosterone concentrations (*P* = .42), and 1.2 years earlier with higher free testosterone concentrations (*P* = .05) but 0.2 years later in girls with higher DHEA-S concentrations (*P* = .59) compared with girls with lower concentrations of these androgens.

**Table 2.** Timing of Breast Development According to Androgen Type, Breast Cancer Family History, and Breast Cancer–Specific Distress Using Weibull Survival Models

Androgen	Hazard Ratio (95% CI)		
	Model 1^a^	Model 2^b^	Model 3^c^
DHEA-S (n = 32)	4.63 (1.46-14.73)	3.69 (1.06-12.89)	6.50 (1.92-21.95)
Breast cancer family history	NA	1.17 (1.01-1.35)	NA
Breast cancer–specific distress	NA	NA	0.22 (0.04-1.12)
Androstenedione (n = 36)	10.83 (2.71-43.31)	16.18 (3.69-70.96)	17.52 (3.58-85.81)
BOADICEA	NA	1.36 (1.15-1.61)	NA
Breast cancer–specific distress	NA	NA	0.32 (0.07-1.50)
Total testosterone (n = 36)	9.60 (2.62-35.26)	14.32 (3.53-58.09)	9.90 (2.65-36.90)
Breast cancer family history	NA	1.37 (1.15-1.63)	NA
Breast cancer–specific distress	NA	NA	0.70 (0.19-2.58)
Free testosterone (n = 36)	4.71 (1.44-15.45)	6.30 (1.77-22.43)	4.79 (1.45-15.81)
Breast cancer family history	NA	1.32 (1.13-1.55)	NA
Breast cancer–specific distress	NA	NA	1.14 (0.32-4.13)

Abbreviations: BOADICEA, Breast and Ovarian Analysis of Disease Incidence and Carrier Estimation Algorithm; DHEA-S, dehydroepiandrosterone sulfate; NA, not applicable.

^a^ Androgen concentrations were adjusted for interaction between the androgen concentration and age, race/ethnicity, and body mass index.

^b^ Model 1 additionally adjusted for BOADICEA score.

^c^ Model 1 additionally adjusted for breast cancer–specific distress.

**Figure 2.**
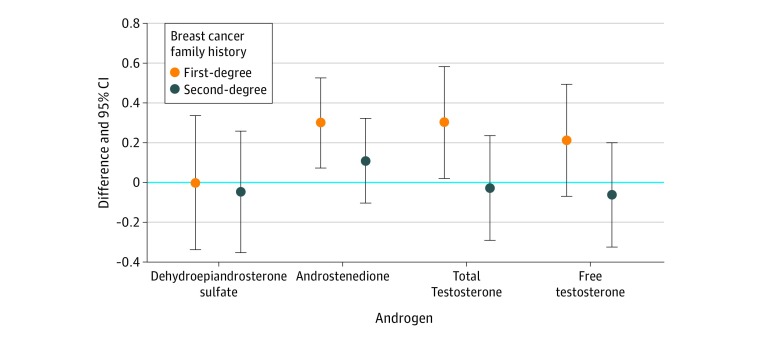
Differences in Serially Measured Androgen Concentrations in Lessons in Epidemiology and Genetics of Adult Cancer From Youth (LEGACY) Girls (Aged 6-17 Years) With vs Without First- and Second-Degree Breast Cancer Family History, Adjusted for Age and Body Mass Index

Compared with no BCFH, a first-degree, but not second-degree, BCFH was associated with 240% higher geometric mean concentrations of androstenedione (no BCFH, 0.49 ng/mL vs first-degree BCFH, 1.8 ng/mL vs second-degree BCFH, 1.6 ng/mL; *P* = .01), 10% higher concentrations of total testosterone (12.7 ng/dL vs 14.0 ng/dL vs 13.7 ng/dL; *P* = .01), and 92% higher concentrations of free testosterone (1.3 pg/mL vs 2.5 pg/mL vs 0.3 pg/mL; *P* = .14) (Figure 2). Percentage differences were calculated from the above means using the following formula:

([|Degree of BCFH − No BCFH|]/No BCFH)100.

We found no difference in DHEA-S concentrations between BCFH-positive and BCFH-negative girls (no BCFH, 41.7 μg/dL; first-degree BCFH, 42.7 μg/dL; and second-degree BCFH, 40.8 μg/dL; *P* = .99). The DHEA-S concentration was higher in girls with breast cancer–specific distress, both unadjusted and adjusted for BCFH (models 2 and 3 in Table 3). This result was specific to DHEA-S and not the other androgens. (To convert androstenedione to nanomoles per liter, multiply by 0.0349; total testosterone to nanomoles per liter, multiply by 0.0347; free testosterone to picomoles per liter, multiply by 3.47; and DHEA-S to micromoles per liter, multiply by 0.027).

**Table 3.** Differences in Androgen Concentrations in 104 Study Participants According to BCFH and Breast Cancer–Specific Distress Using Mixed-Effects Restricted Maximum Likelihood Regression Models

Androgen	β (95% CI)		
	Model 1^a^	Model 2^b^	Model 3^c^
DHEA-S, μg/dL			
First-degree BCFH	0.03 (−0.37 to 0.43)	NA	0.02 (−0.38 to 0.41)
Breast cancer–specific distress	NA	0.12 (0.01 to 0.23)	0.12 (0.01 to 0.23)
Androstenedione, ng/mL			
First-degree BCFH	0.37 (0.12 to 0.61)	NA	0.36 (0.12 to 0.61)
Breast cancer–specific distress	NA	0.04 (−0.12 to 0.20)	0.04 (−0.12 to 0.19)
Total testosterone, ng/dL			
First-degree BCFH	0.39 (0.13 to 0.66)	NA	0.39 (0.13 to 0.65)
Breast cancer–specific distress	NA	0.07 (−0.09 to 0.23)	0.07 (−0.09 to 0.22)
Free testosterone, pg/mL			
First-degree BCFH	0.33 (−0.005 to 0.67)	NA	0.32 (−0.01 to 0.65)
Breast cancer–specific distress	NA	0.13 (−0.07 to 0.33)	0.13 (−0.07 to 0.33)

Abbreviations: BCFH, breast cancer family history; DHEA-S, dehydroepiandrosterone sulfate; NA, not applicable.

SI conversion factors: To convert DHEA-S to micromoles per liter, multiply by 0.027; androstenedione to nanomoles per liter, multiply by 0.0349; total testosterone to nanomoles per liter, multiply by 0.0347; and free testosterone to picomoles per liter, multiply by 3.47.

^a^ Included BCFH adjusted for age and body mass index.

^b^ Included breast cancer–specific distress adjusted for age and body mass index.

^c^ Included BCFH and breast cancer–specific distress adjusted for age and body mass index.

## Discussion

Using a cohort enriched with BCFH, we found that higher serum androgen concentrations were associated with earlier breast development. These results support another prospective study^4^ of 55 German girls, unselected for BCFH, reporting urinary androgens to be associated with earlier breast development. Serum concentrations of testosterone were detectable up to 12 months, androstenedione up to 18 months, and DHEA-S up to 30 months before the onset of breast development in another longitudinal cohort of 252 girls from the United States (<10% BCFH positive).^25^ Our results confirm and extend previous findings, suggesting that androgens are associated with earlier puberty in both BCFH-positive and BCFH-negative girls.

The drivers of elevated prepubertal androgens are unknown. Although age at adrenarche (the activation of the hypothalamic-pituitary-adrenal [HPA] axis) is thought to be consistent across populations, it has been suggested that DHEA-S, the most abundant adrenal androgen, is elevated in prepubertal girls who were born small, were overweight, or experienced stressful life events.^26^ Before puberty, androgens are primarily produced along the HPA axis, also known as the stress axis. Stressors may activate the HPA axis prematurely, increasing the pool of androgens, which are then converted peripherally into estrogens to promote puberty.

Our findings support the hypothesis that the elevated androgen concentrations in BCFH-positive girls may be limited to girls with a first-degree BCFH. Because the production and metabolism of androgens differ before, during, and after puberty and because the stress response system is programmed during puberty, both psychosocial and genetic factors may explain elevated androgens in BCFH-positive girls. In an earlier study, BCFH-positive mothers and daughters reported greater breast cancer–specific distress compared with BCFH-negative mothers and daughters.^22^ If replicated, it is plausible that early-life stress associated with a mother diagnosed with breast cancer may activate the HPA axis prematurely, thereby producing DHEA-S, which would explain our finding that breast cancer–specific distress was specific to DHEA-S and not the other androgens. Dehydroepiandrosterone sulfate is metabolized into androstenedione and testosterone; when puberty begins, the hypothalamic-pituitary-ovarian axis is activated and the ovary starts to contribute 25% to 50% of androstenedione and testosterone in circulation. During puberty the cross-talk between the hypothalamic-pituitary-ovarian and HPA axes may result in differential intracellular metabolism of DHEA-S into downstream androgens by 3β- and 17β-hydroxysteroid dehydrogenase enzymes.^27^

Whether the production and metabolism of androgens is fixed after puberty, which has been noted for stress-induced adrenal glucocorticoid secretion, or can continue to change after puberty is yet to be determined. Emerging evidence from animal models supports the association of stressful environments with steroid hormone production by showing that both physical and social stress can influence glucocorticoid and androgen production.^28^ Furthermore, these models suggest that social stress in adolescence is associated with androgen trajectories, with future implications for disease risk.^29^

### Limitations

Our study was limited by a small sample size, especially in our prospective subset, which examined androgen concentrations and the timing of breast development. Previous studies^25^ have shown that androgen concentrations increase from 18 to 30 months before the onset of puberty; therefore, the prospective study design required the least number of assumptions for statistical analysis. However, to increase our sample size, we also performed a cross-sectional analysis that included girls whose hormone concentrations were measured after the onset of puberty. These measurements were less informative in answering our question as to whether elevated hormone concentrations are associated with earlier breast development. The larger cross-sectional analysis yielded similar results for all of the androgens except DHEA-S. Another limitation was that both the prospective and cross-sectional samples were too small to test for interactions between androgen concentrations and BCFH and breast cancer–specific distress. Thus, larger sample sizes are needed to replicate our findings that prepubertal androgen concentrations are associated with altered timing of breast development and to test whether this association differs in those girls with a BCFH. A larger sample size with more repeated measures will also allow us to address whether androgen concentration trajectories are stable before, during, or after the pubertal transition.

## Conclusions

Epidemiologic studies have reported that higher prediagnostic androgen concentrations are associated with both premenopausal and postmenopausal breast cancer.^2,3^ Women with familial and early-onset breast cancer are largely considered genetically high-risk groups. Our study suggests that girls with and without a BCFH may differ in androgen concentrations as well. Larger prospective studies of the drivers of androgen concentration trajectories during adolescence, when the HPA and hypothalamic-pituitary-ovarian axes are maturing, may be relevant to understanding the increase in breast cancer incidence in women younger than 40 years.^30^
